# Biophilia: Does Visual Contact with Nature Impact on Health and Well-Being?

**DOI:** 10.3390/ijerph6092332

**Published:** 2009-08-31

**Authors:** Bjørn Grinde, Grete Grindal Patil

**Affiliations:** 1 Norwegian Institute of Public Health, PO Box 4404 Nydalen, 0403 Oslo, Norway; 2 Department of Plant and Environmental Sciences, Norwegian University of Life Sciences, Box 5003, N-1432 Ås, Norway; E-Mail:grete.patil@umb.no

**Keywords:** biophilia, discord, quality of life, health, evolutionary psychology, plants, indoor environment

## Abstract

It is concluded that an environment devoid of Nature may act as a “discord”, i.e., have a negative effect. While the term mismatch is used for any difference between present living conditions and the environment of evolutionary adaptation, discords are mismatches with a potentially undesirable impact on health or quality of life. The problem is partly due to the visual absence of plants, and may be ameliorated by adding elements of Nature, e.g., by creating parks, by offering a view through windows, and by potted plants. The conclusion is based on an evaluation of some fifty relevant empirical studies.

## 1. Introduction

The hypothesis that humans have an inherent inclination to affiliate with Nature has been referred to as biophilia [1,2]. Biophilia implies affection for plants and other living things. Cities and indoor environments are dominated by manmade objects; the question is whether the concomitant depletion of natural elements has a negative impact on the human mind.

In most cultures, both present and past, one can observe behavior reflecting a fondness for Nature. For example, tomb paintings from ancient Egypt, as well as remains found in the ruins of Pompeii, substantiate that people brought plants into their houses and gardens more than 2,000 years ago [3]. Moreover, in most cities, trees are planted and parks established in order to improve the environment. A tendency to add elements of Nature seems to be a universal human feature; evident wherever manmade surroundings tend to remove humans from a natural setting, and where the people are sufficiently affluent to afford doing something about it. The behavior is, presumably, a response to the biophilic quality of the human mind [4].

The first hospitals in Europe were infirmaries in monastic communities where a garden was considered an essential part of the environment in that it supported the healing process [5]. Since then the connection between greenery and either therapeutic or preventive medicine has gradually been outmoded, partly due to the advance of medical science and the concomitant technical approaches to healing. Over the last decades, however, considerable research has been carried out looking at the effects of being in Nature, and of adding plants to otherwise sterile environments. To the extent that the results are positive, the idea that access to nature can aid healing, or help prevent ailments, may eventually be incorporated into evidence based medicine.

Adding elements of Nature to living spaces can presumably induce positively valued changes in cognition and emotion, which again may impact on stress level, health and well-being. In order to allocate resources for the purpose of creating more natural environments, it is important to assess what sort of return can be expected. Here we review a range of current data, focusing primarily on recent work published in established scientific journals. Some fifty empirical studies were examined with the following aims: One, to verify whether the biophilia hypothesis has merit; two, to suggest what sort of influence the presence of plants may have on the human mind; and three, to evaluate to what extent adding elements of nature can compensate for visits to the outdoors and thereby be used as a preventive measure to improve health and well-being. Although plants may enhance the environment in several ways, including improved air quality and the addition of fragrance, we here focus on the visual impact.

## 2. Theoretical Perspectives

Humans, like any other species, have been shaped by the forces of evolution. The term *Environment of Evolutionary Adaptation*, or EEA, is used to denote the qualities of the environment humans are adapted to live in [6,7]. Obviously this environment comprised a closer presence of Nature compared to what most people experience today. Plants were of crucial importance for survival during most of our evolutionary history; as a food resource, for shelter, and as an indicator of water. On a purely theoretical ground, one would expect the presence of plants, as an integral part of the human EEA, to have had an impact on the evolution of the brain. We are presumably adapted to live in a green environment.

Deviations from the way of life for which we are genetically designed have been referred to as *mismatches* [8]. Some mismatches are beneficial, such as sleeping on a mattress instead of on the ground, while others may contribute to disease or reduce life quality. The word *discord* is used for mismatches that have a negative impact; i.e., they cause some form of “stress”, at least in susceptible individuals [9].

Zoological gardens illustrate the role of discords. Zoo keepers need expertise as to what sort of conditions one ought to provide the various species of animals. As a rule of thumb the ideal is to approach as close as possible the EEA of the species in question; i.e., to offer the type of conditions that the species have in the wild. Refraining from this rule easily leads to animals that show inappropriate behavior such as hurting themselves and refusing to mate or eat. Obviously it is impossible to offer the exact EEA within the confinement of a zoo, thus the focus is on avoiding the more troublesome discords.

Modern societies can be construed as “zoological gardens” in that the environment necessarily is different from the EEA. A relevant step towards improving the situation is to avoid discords by creating an environment that approaches as much as possible the EEA. A constructive strategy is to suggest candidate discords by comparing present living with assumptions about the environment humans are adapted to live in, and subsequently assess these putative discords by empirical research. The implications, as to the presence of plants, is that although the absence of natural elements is an obvious mismatch, research is required to decide to what extent it is also a discord.

Although any organ or bodily function can suffer from discords, the human brain appears to be particularly vulnerable—due to its complexity, the fact that it requires substantial maturation after birth, and that the maturation takes place in response to environmental stimuli. This vulnerability presumably helps explain why mental disorders are one of the main health problems of Western societies [10]. Thus, to the extent that a lack of natural elements is a discord, one would expect that a closer association with nature should improve psychological health. Most of the research related to biophilia has focused on positive effects of associating with plants rather than negative, i.e., discord, effects of removing greenery. According to the concept of discords, a positive effect suggests that those who presently obtain a suboptimal dose of exposure to plants have a concomitant reduced life quality. Current statistics of mental health does not contradict this model.

Most studies dealing with psychological benefits of Nature are within the field of environmental psychology, and are typically based on theories of restorative effects. Restoration, in this context, implies the process of regaining psychological, social and physical capacity [11]. One theory suggests that the visual environment is important for stress recovery and that stress reduction is faster in Nature compared to urban environments [12,13]. It is argued that stress activation has evolved through evolution as a strategy to deal with situations that threatens well-being. Too much stress may lead to various ailments, including anxiety related disorders [14]. A visual presence of plants may be one such stress-reducing factor as affective responses to visual stimuli deemed aesthetic may release tension. Beauty has been defined as visual input that gives pleasure to the mind, thus aesthetics offer per definition a positive experience. A theoretical examination of aesthetic values points towards the importance of elements reflecting Nature; such as complexity, choice of colors, perspective and balance [15]. In other words, Nature itself may offer potent aesthetic stimuli.

The Attention Restoration Theory offers an alternative way of explaining psychological benefits of Nature [16]. Directing attention to demanding tasks and dealing with disturbing environmental factors may lead to mental fatigue. On the other hand, environments that provide a possibility for more effortless attention offer an opportunity to restore mental capacity. Surroundings dominated by elements of Nature are thought to be restorative.

Although it would be useful to understand how the visual presence of plants can have a positive effect on well-being and health, one should be open for the possibility that the natural environment influences subconscious parts of the brain in ways that cannot easily be described. Objects within the field of vision may in fact exert an influence even if the conscious brain does not recognize their existence. The classical example is the response evoked by a twig on the ground if it remotely resembles a snake: The fear is initiated prior to any visual inspection of the twig. Similarly, plants may impact on brain processes through unconscious mechanisms even when they are not the object of focus. The absence of plants may suggest an “unnatural”, and thus potentially unsafe, environment.

Non-visual aspects of adding plants to the environment may also play a role, for example fragrance [17], or improving acoustics [18]. Moreover, effects on health can be conveyed by the way plants influence the microclimate, i.e., by improving humidity and purifying the air [19,20]. The present review will focus on visual aspects. Although empirical data offer clues as to possible advantages of associating with Nature, it should be noted that in most cases there is limited information as to how the effects are elicited.

## 3. Empirical Studies on Outdoor Environment

Over the past decades, an increasing number of studies have documented that experiences in, or of, Nature can be beneficial for human health and well-being. The issue has been reviewed in a report for the Health Council of the Netherlands [21], which concludes that there is a positive link between health indicators and living close to Nature.

More specifically, contact with Nature has been reported to have psychological benefits by reducing stress [12,22], improving attention [16], by having a positive effect on mental restoration [23–25], and by coping with attention deficits [26,27]. In addition to mental advantages, there appear to be direct physical health benefits [28], such as increased longevity [29], and self-reported health [30,31]. As might be expected, the availability of Nature correlates positively with health [32]. Benefits have been associated with various types of Nature experiences, including true wilderness [33,34], neighbourhood parks [35,36], gardens [37–39], and natural features around residences [40,41].

The stress reducing effect may be a key element as to the health benefits of Nature. Stress plays a role in the etiology and course of several common health problems, including cardiovascular diseases, anxiety disorders and depression. It is noteworthy that beneficial effects of Nature can occur even upon relatively brief exposure.

A main concern with most of the studies mentioned above is to decipher what is actually causing the benefits. Ulrich [13] points to four possible advantages: One, being in Nature tends to correlate with physical activity, which obviously promotes health. Two, Nature activities often implies socializing, e.g., in the form of walking together or sitting in a park with friends. Building social networks has a well documented potential for improving health. Three, Nature offers temporary escape from everyday routines and demands. The fourth option is the question of to what extent the interaction with Nature itself has an appreciable impact on the mind; in other words, is there an extra benefit of performing these tasks in a natural environment, or can the physical and social advantages alone explain the observed benefits?

The idea that being in Nature may improve health has led to organized activities referred to as therapeutic horticultural (for a review, see [42]). The term typically implies that a group of people comes together to do gardening or in other ways interact with or care for plants. Therapeutic horticultural activities have apparently had some success, primarily for people with mental health problems or learning difficulties, although empirical data is limited [43].

If Nature itself is responsible for some of the advantages, the next question is how to explain this effect? Again there are at least three options: One, the air may be more healthy in that it contains less air pollutants and more humidity; two, the plants may emit fragrances that humans find pleasant or react to in various ways [17,44]; or three, which is the main subject for the present review, the visual experience of plants makes a difference. As will be discussed below, some reports contain data relevant for singling out the potential of the latter option.

One approach relevant to the task of distinguishing between visual and non-visual effects is to consider the outcome of simply viewing Nature through a window or seeing pictures of Nature. To the extent that looking at Nature makes a difference, the other possible explanations can normally be ruled out. It has been reported that viewing natural landscapes provides psychological and health benefits, including a reduction in stress [12,13,45]. Having a hospital window with a view has been shown to improve healing, reflected in both the level of pain medication and the speed of recovery after surgery [48,49]. In reviewing this issue, Velarde *et al.* [50] found that natural landscapes have a consistent positive health effect, while urban landscapes can have a negative effect

To conclude this section, nature appears to have qualities useful for stress relief, mental restoration, and improved mood simply by being consciously or unconsciously “pleasing to the eye”. Although there are several other ways in which the availability of plants can contribute to health, the visual aspect is presumably sufficient to offer some advantage.

## 4. Empirical Studies on Indoor Environment

The next question is whether adding elements of Nature, in the form of plants or other items resembling Nature, to indoor environments offers some of the advantages of outdoor nature. This is a relevant question as we spend a major part of our time indoors [51].

It has been shown that office employees seem to compensate for lack of window view by introducing indoor plants or pictures of Nature [52]. An ensuing question is whether the plants or pictures improve performance, health, or well-being for the employees. In the same study population it was found that having a view to plants from the work station decreased the amount of self-reported sick leave [53].

Experimental studies on psychological benefits of indoor plants have recently been reviewed in a report including more than twenty studies [54]. Most of these studies concern people in settings reflecting everyday life, such as the workplace, students at school, or patients in hospitals. Some studies were more experimental in Nature, typically recruiting college students as subjects for testing the effect of plants in the laboratory. Almost all of the studies had a no-plant control condition, but otherwise they showed considerable variation in experimental manipulations, both quantitatively (e.g., number of plants) and qualitatively (e.g., a distinction between flowering and non-flowering foliage, size, shape and plant species). The duration of exposure to plants also varied, from minutes in laboratory studies up to a year in workplace settings. The measured outcomes reflected practical concerns of the research, and included task performance, affect, physiological arousal, pain perception, health and discomfort symptoms, social behavior, and room evaluations. Some studies found beneficial effect(s), while others did not, or only found them for some groups. None of the studies reported any significant negative outcome associated with the presence of plants.

Several studies indicated that indoor plants improve the attractiveness of a room [55–58]. Dijkstra *et al.* [58], for example, found that by showing photos of hospital rooms with or without plants, those with plants reduced self-reported stress. Other studies also indicate lower stress level when adding plants to a windowless work environment [22,59].

The biophilia hypothesis might suggest an impact of plants on emotional states; however, several studies have failed to find any consistent impact [56,60–62]. Some studies, using mood scales including several items, found significant differences, but only on particular items [57,59,63]. Adachi *et al.* [57] even reported possible negative effects of plants on annoyance and temper. A couple of reports suggested gender differences in that women, particularly those with a relatively high level of preinduced stress, had the most benefit [17,44].

The idea of a stress-reducing effect also inspired experiments concerned with pain and recovery from disease [63–66]. One starting point for these studies was the idea that the pleasant and attention holding (i.e., positively distracting) properties of plants might keep a person from focusing on pain. All the studies concluded that the subjects had better tolerance for pain with than without plants present. One report [64] suggested that flowering plants have more positive effects on pain tolerance and distress than non-flowering plants. Lohr and Pearson-Mims [63] observed an effect on pain tolerance, apparently due to more than just a distracting quality of plants.

Other experiments have looked at the effect of plants on task performance or self-reported alertness [56,59,60,62]. The idea is that the presence of indoor plants may help restore attention by relaxing the subjects and help them recover from mental fatigue. Positive effects of plants were reported, although the results are somewhat ambiguous. One report found that performance on a letter identification task decreased with the presence of a larger number of plants, which was taken to suggest that fascination with plants may interfere with the focus on the task at hand [56].

A decrease in health complaints, such as tiredness and coughing, has been reported in office and hospital workers when plants were added to the work environment [67,68]. Similar findings on conceived health and level of discomfort were observed in school children [68]. The authors ascribe the positive outcome in these experiments to either an improvement in air quality, or that a more pleasant visual environment affected the amount of health complaints.

It is worth mentioning that plants may be viewed as one among many types of aesthetic features added to enhance indoor environments. A study by Lohr and Pearson-Mims [63], however, suggests that plants may have advantages. They found that plants had greater attention holding power and gave greater relief from pain compared to other aesthetic objects such as a designer lamp or an abstract picture. The room with plants was also perceived as more cheerful, pleasant, and inviting.

As in the case of the outdoor studies, it is not obvious that the indoor results reflect solely the visual presence of plants. It is difficult to exclude an effect of fragrance or of air quality. However, it seems fair to assume that visual impact is an important factor.

## 5. Discussion

Taking all the reviewed evidence into account, the idea that interacting with Nature can offer positive effects on health and well-being seems to be reasonably well substantiated. Thus, the biophilia hypothesis has merit. The evidence includes studies on outdoor activities, therapeutic use of Nature, having a view of Nature (either actual Nature or in pictures), and adding plants to indoor environments. Moreover, the notion that part of the effect is mediated through visual contact with plants also appears to be substantiated. The above statement is based on empirical data, but supported by theoretical expectations, which suggest that the absence of Nature is a potential discord. The latter point has been raised recently by Richard Louv [69], who use the term *nature-deficit*, and suggests that the increase in prevalences of conditions such as obesity, attention disorders, and depression is partly due to a decrease in the degree children are exposed to Nature.

Biophilia may be described as a vague preference for having a natural environment as a consequence of our evolutionary history. As such, one would expect that plants are agreeable, and that the absence of greenery is sensed, possibly unconsciously, as a stress factor. In other words, the presence of plants can impact on the human mind. Biophilia, however, is probably not an attribute with a strong penetrance. Thus the relationship between humans and plants is likely to be shaped to a large extent by cultural factors and individual peculiarities [47].

On a theoretical basis, it should be expected that if plants in a natural setting have an impact, so would indoor greenery. However, one might also expect that disconnected, potted plants are less potent than outdoor Nature. The overall trend in the literature appears to support this contention. In their review, Bringslimark *et al.* [54] focused on the benefits of indoor plants. They concluded that although some findings recurred, such as enhanced pain management with plants present, the mixed results from the studies suggest that more research is needed in order to define possible effects. None of the studies reported obvious negative effects. It might be argued that if there was no effect, an equal number of studies would be expected to find negative as positive correlates between health parameters and the presence of plants. On the other hand, publications are liable to the bias of preferential reporting of positive results. It is not possible to know how many trustworthy neutral or negative findings that are not published, but the fact that several articles reported absence of effect indicates that both types of results would be publishable.

One problem in detecting possible effects is that most studies, for practical reasons, span a short time-period. Some only look at brief exposure to plants, while others may follow subjects for a year or so. To the extent that the absence of plants is a discord, one might expect that the consequences are more likely to be apparent over a life time. Moreover, although the therapeutic or preventive potential of plants is likely to be limited, as the indoor environment is the daily setting for a majority of the present population, even minor effects of adding plants can add up to a substantial decrease in the health burden on a global scale.

The positive effect of having a view from the window may be related more to the perceived openness than to any particularities of the vista. Velarde *et al.* [50] addressed this issue and concluded that seeing open water is better than open city landscapes, but that green landscapes offered the best effect. In this context, it should, however, be mentioned that green spaces perceived to be unmanaged may have an adverse effect in the cities by causing an increased anxiety for crime [70].

Some studies reported differences in the response to plants depending on gender [17,44,61,62]. Although the results were somewhat mixed, there seemed to be a tendency for women to respond stronger to plants than men. On a theoretical ground one might expect that women take more interest in plants due to differences in activities during the formative period of human evolution; that is, women were supposedly more involved in gathering plants as food, while men were more tuned towards hunting. However, the difference may also be due to cultural bias; for example, in Western societies it has traditionally been the task of women to care for the home, which will typically include both garden and indoor plants.

There seems to be a current trend towards a love for TV and computer screens rather than for nature, in that people use the former more and the latter less [71,72]. Although indoor plants may ameliorate some of the negative effects of this trend, it can hardly be more than a substitute for experiencing real Nature outdoors.

The biophilia trait can be reinforced or subdued by individual learning. It seems likely, however, that even in individuals who do not express any appreciation for plants and nature, the lack of nature can have a negative effect. Moreover, although the demonstrated effects are not overwhelming, the cost of making nature available, if only as potted plants, is neither prohibiting. In other words, it seems worthwhile to encourage interaction with plants, both outdoor and indoor, as this is likely to be a useful environmental initiative with a sound cost-benefit profile.
